# Typical Facial Lesions: A Window of Suspicion for Progressive Disseminated Histoplasmosis—A Case of Asian Prototype

**DOI:** 10.1155/2016/2865241

**Published:** 2016-09-26

**Authors:** Prasan K. Panda, Siddharth Jain, Rita Sood, Rajni Yadav, Naval K. Vikram

**Affiliations:** ^1^Department of Internal Medicine, All India Institute of Medical Sciences, New Delhi 110029, India; ^2^Department of Pathology, All India Institute of Medical Sciences, New Delhi 110029, India

## Abstract

Histoplasmosis is caused by a dimorphic fungus* Histoplasma capsulatum* in endemic areas, mainly America, Africa, and Asia. In India, it is being reported from most states; however, it is endemic along the Ganges belt. We report a case of an apparently immunocompetent male who presented with 3-month history of fever, cough, and weight loss with recent onset odynophagia and had hepatosplenomegaly and mucocutaneous lesions over the face. The differential diagnosis of leishmaniasis, tuberculosis, leprosy, fungal infection, lymphoproliferative malignancy, and other granulomatous disorders was considered, but he succumbed to his illness. Antemortem skin biopsy and bone marrow aspiration along with postmortem liver, lung, and spleen biopsy showed disseminated histoplasmosis. This case highlights the need for an early suspicion of progressive disseminated histoplasmosis in the presence of classical mucocutaneous lesions even in an immunocompetent patient suffering from a febrile illness. Cure rate approaches almost 100% with early treatment, whereas it is universally fatal if left untreated.

## 1. Introduction

Classical histoplasmosis, also known as Darling disease, was first discovered in 1906 [1]. It is an endemic mycosis, caused by two species known to be pathogenic to man (*H. capsulatum* var.* capsulatum* and* H. capsulatum* var.* duboisii*).* H. capsulatum* is highly endemic in North America along the rivers Ohio and Mississippi, but Southeast and Southern Asia have focal endemicity, which is underrecognized due to the low awareness of the disease, misdiagnosis of the disease often as tuberculosis or leishmaniasis, and lack of proper diagnostic facilities [2]. “Asian histoplasmosis” as proposed differs from the American or African type, in having more mucocutaneous manifestations and a propensity for acute adrenal insufficiency, but the latter fact is disputed in many recent studies [3]. In India, the first case of histoplasmosis was reported in 1954, following which many case reports, two successive systematic reviews, and three large hospital based retrospective studies have been published in the literature [4–6]. A majority of cases have been reported from the eastern parts of the country, especially along the belt of Ganges and Brahmaputra, which may be related to the climate, humidity level, and soil characteristics. Due to migration and increased urbanization, cases are being reported from all over the country. Clinical suspicion should be high to diagnose cases in nonendemic areas.

This fungus grows in soil enriched with bird droppings, reaches human alveoli through inhalation, and causes varied clinical presentations ranging from self-limiting flu-like illness or acute or chronic pulmonary histoplasmosis to progressive disseminated histoplasmosis, depending on the quantity of antigen exposure and immune status of the individual [6]. All organs can be involved during the process of dissemination, but the reticuloendothelial system, skin, adrenals, gastrointestinal tract, and lungs are the most commonly involved sites [7]. Henceforth, skin may act as a window for early diagnosis of disseminated histoplasmosis.

We report an immunocompetent patient with disseminated histoplasmosis in whom an early suspicion of the disease may have improved the prognosis.

## 2. Case Presentation

A 50-year-old male, resident of an area in the Ganges belt of Uttar Pradesh, India, presented with complaints of cough with scanty whitish expectoration, intermittent low grade fever, generalized weakness, and weight loss for three months. He also complained of abdominal pain, nonbilious vomiting, and progressive swelling over both legs for one month. Additionally, he noticed appearance of gradually progressive nonpruritic skin eruptions over the face and painful oral ulcers with odynophagia for the last three weeks. He also reported a history of melena for the last 10 days. There was no history of chest pain, hemoptysis, breathlessness, or urinary complaints. He was a tea vendor by occupation with no known prior illness.

**Figure 1:**
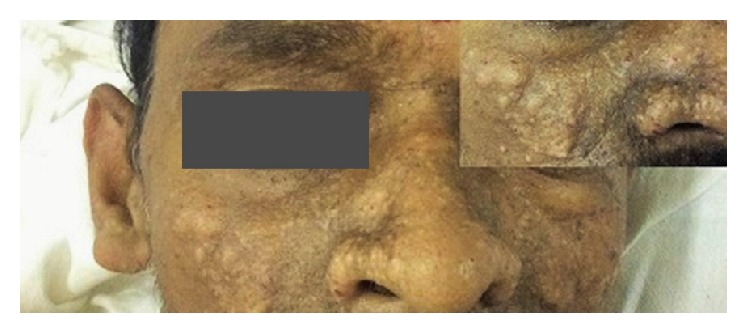
Face monograph showing skin colored, nonpruritic, nontender, papulonodular lesions (few umbilicated). Inset showing close-up view of right side facial lesions. Gray bar was used to mask face recognition.

On examination, he was conscious and hemodynamically stable. There was moderate to severe pallor, mild icterus, and bilateral pedal pitting-edema. He had oral nonaphthous ulcers with bleeding spots and multiple skin colored papulonodular lesions (with few showing central umbilication) over the face and bilateral ear lobes (Figure 1). There were no peripheral lymph nodes palpable. Cardiorespiratory examination was unremarkable except for occasional basal crackles. Abdominal examination revealed 4 cm, nontender, firm hepatomegaly and 3 cm, nontender, firm splenomegaly below the costal margin. Examination of other systems was unremarkable.

The different diagnoses considered at admission were post-kala-azar dermal leishmaniasis, disseminated tuberculosis, leprosy, invasive fungal infection, lymphoproliferative malignancy, and other granulomatous disorders.

The blood picture revealed Hb of 86 g/L, platelet count of 49 × 10^9^/L, WBC count of 7.1 × 10^9^/L with neutrophilic predominance (80%), and ESR of 50 mm/hr. Liver function tests (LFT) showed total bilirubin of 29.08 *μ*mol/L (direct fraction, 23.95 *μ*mol/L), AST of 0.83 *μ*kat/L, and ALT of 0.60 *μ*kat/L. The serum alkaline phosphatase (ALP) was very high (41.00 *μ*kat/L), but LDH was in the upper normal range (4.43 *μ*kat/L). There was hypoproteinemia (serum total proteins 45 g/L) with reversal of albumin/globulin ratio (1.6 : 2.9). Prothrombin time and thrombin time were deranged, and D-dimer levels were found to be very high. Kidney function tests and urine examination were normal. In view of fever, hepatosplenomegaly, bicytopenia, and deranged LFT, a possibility of secondary hemophagocytic lymphohistiocytosis was also considered. Serologies for kala-azar, HIV, and viral hepatitis B and hepatitis C were negative.

Chest X-ray was essentially normal. Contrast-enhanced CT scan of the thorax and abdomen revealed hepatosplenomegaly with a splenic infarct, multiple calcified mediastinal nodes, fibrotic opacities in the bilateral lung apices (suggesting sequelae of old infection), minimal bilateral pleural effusion and ascites, and no lymphadenopathy. The pleural fluid was exudative with lymphocytic predominance. GeneXpert MTB/RIF of the fluid was negative. Punch biopsy was taken from the skin lesions on the face. A bone marrow biopsy was subsequently done. An upper GI endoscopy was performed which showed features suggestive of diffuse gastritis with punctate submucosal hemorrhages but biopsy could not be taken due to increased risk of bleeding.

**Figure 2:**
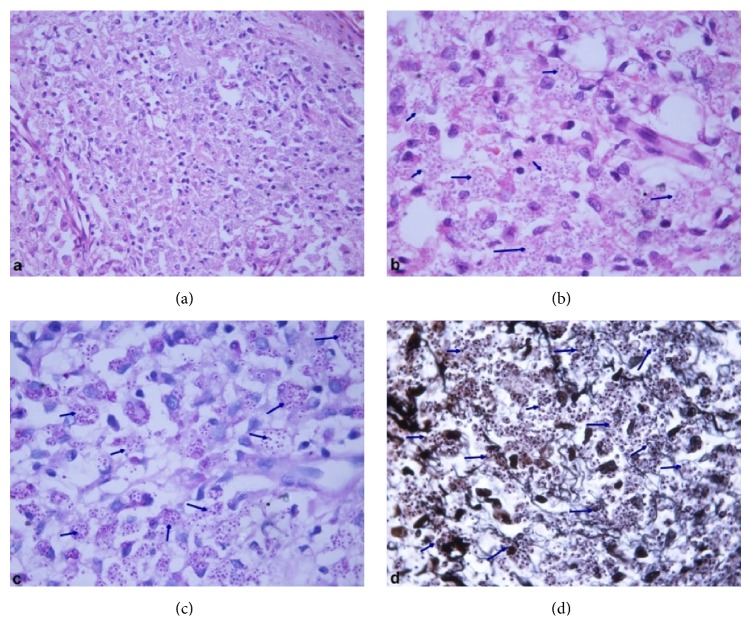
Skin biopsy shows a dense dermal histiocytic infiltrate with the presence of numerous intracellular spherical organisms surrounded by a halo in histiocytes, hematoxylin and eosin (H&E): (a) ×400 and (b) ×1000. These organisms stained positive with periodic acid Schiff (c) ×1000 and silver methenamine (d) ×1000 stains. All are morphologically compatible with* Histoplasma* as focused with arrows.

**Figure 3:**
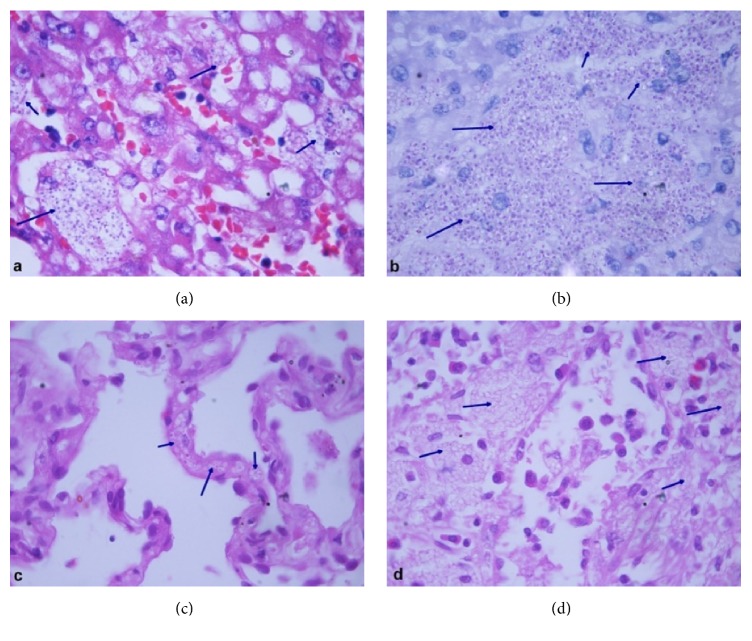
Liver biopsy shows numerous spherical organisms in the sinusoidal macrophages and Kupffer cells, (a) H&E ×1000, which stained positive with periodic acid Schiff stain (b) ×1000. Similar organisms are also seen in interstitial histiocytes in lung, (c) H&E ×1000, and splenic macrophages, (d) H&E ×1000. All are morphologically compatible with* Histoplasma* as focused with arrows.

The patient was managed with broad spectrum intravenous antibiotics, intravenous albumin, multivitamins, platelet concentrates, and packed RBC transfusions while awaiting the aforementioned biopsy reports. The patient developed altered sensorium and required endotracheal intubation and mechanical ventilation. Repeat LFT showed rising bilirubin levels with deranged PT/INR and aPTT suggestive of disseminated intravascular coagulation. A day later, the patient developed shock requiring inotropic support. Meanwhile, the provisional report of bone marrow aspirate indicated possibility of histoplasmosis. Skin biopsy showed a dense dermal infiltrate composed mainly of histiocytes and a few lymphocytes (Figure 2). The histiocytes showed numerous intracellular 2–5 *μ*m oval to round organisms surrounded by a halo, which stained positive with periodic acid Schiff and silver methenamine stains, thus confirming* Histoplasma*. He was thus started on conventional intravenous Amphotericin-B at a dose of 1 mg/kg/day. However, on the same day, he succumbed to his illness. Postmortem biopsies of lung, liver, and spleen were taken (with consent of the relatives) all of which showed numerous intracellular spherical organisms in the histiocytes present in the lung interstitium, sinusoidal Kupffer cells of liver, and splenic macrophages, morphologically compatible with* Histoplasma* (Figure 3). The final diagnosis of chronic progressive disseminated histoplasmosis, involving liver, spleen, lung, GIT, bone marrow, and skin, was made.

## 3. Discussion

Histoplasmosis, caused by the dimorphic fungus* H. capsulatum*, has a spectrum of manifestations ranging from asymptomatic disease (>99% of cases) to progressive disseminated histoplasmosis (PDH) depending upon the intensity of exposure and immune status of the patient [7]. Further, onset of PDH may be as follows [8]:

- Acute, seen in infants or immunocompromised people with certain risk factors, having high mortality where the risk factors for progressive disseminated histoplasmosis are as follows:
  1. Age (infants)
  2. AIDS
  3. Hematologic malignancies
  4. Solid organ transplant
  5. Hematopoietic stem cell transplant
  6. Immunosuppressive agents
    1. Corticosteroids
    2. Tumor necrosis factor antagonists
  7. Congenital T-cell deficiencies
  8. Gamma-interferon receptor deficiency
  9. Hyperimmunoglobulin M syndrome
- Subacute, the most common type with a relentless course and focal lesions in various organs
- Chronic, slowly progressive symptoms of organ involvement in old age and immunocompetent individuals with invariable death if not treated

Our case, in the absence of risk factors, belongs to the category of chronic PDH. The patient was a tea vendor by occupation. His shop was situated under a tree where a large number of birds nested. One possible source of infection could have been exposure to bird droppings. Further, his residence was close to Ganges belt from where cases of* Histoplasma* have been reported frequently [6]. There are not many studies to prove seasonal variation, but one French study showed a peak of infection during a long dry season [9]. Similar to our case that has been symptomatic in July 2014, there was another case presented in July 2012 as reported from Southern India [10].

No single organ is spared as dissemination proceeds but PDH should be considered as a possible differential diagnosis in all cases of fever of unknown origin (FUO), significant weight loss, adrenomegaly, hepatosplenomegaly, lymphadenopathy, or mucocutaneous lesions [11]. After self-limiting acute pulmonary histoplasmosis resolves due to adequate cellular immunity, calcified lung nodules and/or mediastinal lymph nodes remain which may persist for life [8]. Apical pleural thickening is common, but isolated pleural effusion (exudative, mostly hemorrhagic) is uncommon. Pleural effusion is common in a setting of acute pericarditis and is due to host immune response to fungi [12]. Our case had both calcified lymph nodes and exudative pleural effusion without features suggestive of pericarditis. Therefore, there is a high possibility of host immune response leading to the bilateral pleural effusion seen in our case. Lung biopsy depicted* Histoplasma* despite lack of any imaging evidence of acute/chronic pulmonary histoplasmosis, suggesting subtle involvement; however, acute pulmonary infections are not typically associated with a chest X-ray abnormality [7].

Mucocutaneous histoplasmosis is very common in HIV patients and is rarely seen in immunocompetent individuals, but histoplasmosis in Asians has been shown to have higher mucocutaneous involvement in the latter group also. In one large case series having 61 immunocompetent patients, overall mucocutaneous involvement was found to be 36% [5]. Oral involvement in PDH, in the form of painful ulcers, nodules, or wart-like growths involving tongue, palate, gingiva, and oropharynx, (incidence 25–45%), needs to be differentiated from squamous cell carcinoma, hematological malignancies, tuberculosis, other deep fungal infections, or all manifestations of Crohn's disease or chronic traumatic ulcers [13]. In non-HIV patients, skin lesions were observed to be uncommon (9%) in one study [14]. The most common skin lesion is a papule, plaque, pustule, or nodule with or without central umbilication, resembling molluscum contagiosum, acneiform eruptions, or sebaceous hyperplasia [15]. Once detected, these classical mucocutaneous manifestations are hallmark of underlying PDH, especially in immunocompetent individuals like in our case, though localized skin involvement has also been rarely reported [13]. Biopsy of these lesions is a rapid way for diagnosis, preventing delay in administration of life-saving treatment. Our patient may have been diagnosed earlier if skin biopsy was done prior to the admission of the patient to the hospital.

Hematological manifestations like anemia, thrombocytopenia, pancytopenia, or increased ESR have been reported [6]. These are due to granulomatous involvement of bone marrow, secondary hemophagocytic lymphohistiocytosis, or immune mediated destruction of blood cells. Our patient had documented bone marrow involvement with possibly an element of immune thrombocytopenia since large platelets were seen in the peripheral smear. As Subbalaxmi et al. have reported, PDH should be considered in the differential diagnosis of thrombocytopenia with FUO irrespective of the patient's immune status and endemicity of the disease [10].

Gastrointestinal histoplasmosis (GIH) has been found in 70–90% of cases at autopsy but is rarely encountered during life because of minimal clinical symptoms and lack of suspicion [16]. Therefore, an active search through endoscopy should be made especially in patients with suspected PDH with apparently normal immune function, even in the absence of gastrointestinal symptoms. Endoscopy may show superficial mucosal ulceration, deep ulceration with or without perforation, or friable masses with or without obstruction [16]. Colon is the most common site involved in GIH followed by the small intestine [8]. Common symptoms are abdominal pain, diarrhea, nausea, vomiting, tenesmus, or constipation. Hepatomegaly and/or splenomegaly are reported in 30–100% of cases [16]. Our case had gastritis with submucosal hemorrhages and hepatosplenomegaly.

Culture is the gold standard for diagnosis, but histopathology/staining is the investigation of choice before starting treatment due to early reporting. The presence of tiny 2–4 *μ*m spores with a clear zone around the nucleus, inside or outside macrophages or giant cells, visualized with hematoxylin & eosin, periodic acid Schiff, silver methenamine, Giemsa, lactophenol blue stains, or electron microscopy, is a distinguishing feature of* H. capsulatum* differentiating from* Leishmania*,* Penicillium marneffei*,* Cryptococcus neoformans*, and* Candida glabrata* [15]. Antigen detection and antibody serology are rapid diagnostic modalities; however, both have false positive and false negative results due to dependency on factors like quantity of antigen exposure, chronicity of disease, and immune status of patients [7]. One study showed culture positivity rates for lymph node, liver, and spleen biopsy samples, blood, and BAL to be 52.9%, with GI specimen showing a culture positivity rate of 90.9% [17]. Hence, all mucosal lesions identified at endoscopy should be cultured for fungi. This could not be done in our patient due to the high risk of bleeding.

Timely administration of antifungals decreases mortality to less than 25% in PDH, but, without treatment, the probablity of mortality is 80–100% [18]. For severe acute pulmonary histoplasmosis and severe PDH, Amphotericin-B, especially the liposomal formulation, is the drug of choice. For other types, azoles, mainly itraconazole, are sufficient. Relapse rate is high, up to 10–20% in PDH and up to 80% in AIDS associated cases [16].

## 4. Conclusions

1. Mucocutaneous characteristics are pathognomonic of underlying PDH. Early local site biopsy can clinch the diagnosis and skin may be a window to diagnose deep fungal infections.
2. GIH with diffuse gastritis and punctate submucosal bleeding spots is also an important finding. This may act as a window to diagnose PDH if biopsy is possible.
3. One should consider histoplasmosis when symptoms are primarily respiratory (even with apparently normal chest imaging) with secondary dissemination of symptoms and signs. This is because the route of entry of this fungus is via the lungs.
4. PDH is a highly underrecognized fungal infection in India even today. This case is again reminding us of the “Asian type of histoplasmosis.”
5. Lastly, this case reiterates the importance of doing biopsy on time before it is too late.
